# Bernard-Soulier Syndrome: Case Studies From Morocco

**DOI:** 10.7759/cureus.87578

**Published:** 2025-07-09

**Authors:** Fatima Zahra Lfaquir, Hassane Mamad, Khalil Zimi, Souad Benkirane, Masrar Azlarab

**Affiliations:** 1 Central Laboratory of Hematology, Ibn Sina University Hospital, Faculty of Medicine and Pharmacy, Mohammed V University in Rabat, Rabat, MAR

**Keywords:** bernard-soulier syndrome, gpib-v-ix complex, morocco, platelet aggregometry, primary hemostasis

## Abstract

Introduction: Bernard-Soulier syndrome (BSS) is a rare thrombopathy with only a few hundred cases reported in the medical literature. This study aims to highlight the diagnosis of this thrombopathy by platelet aggregometry at the central hematology laboratory of Centre Hospitalo-Universitaire Ibn Sina in Rabat.

Materials and methods: We conducted a retrospective, descriptive study of BSS patients diagnosed in our laboratory over a four-year period, from 2020 to 2024. The study was based on the results of blood count and platelet aggregometry performed on platelet-rich plasma using the APACT 4004 device (LABiTec® Labor BioMedical Technologies GmbH, Ahrensburg, Germany).

Results: Out of 268 platelet aggregation tests, seven patients had an aggregometric profile compatible with BSS. The mean age was 21 years, with a sex ratio of 2.5. Second-degree consanguinity was found in six cases. Clinically, six patients had a history of hemorrhage since early childhood, while only one patient presented with non-traumatic hemarthrosis at an advanced age compatible with acquired BSS. Biologically, all patients had a normal hemostasis profile. Microcytic hypochromic anemia was observed in four cases. All had thrombocytopenia, except in one case where thrombocytosis was observed. Blood smears showed macroplatelets in all patients. Platelet aggregation showed a normal response to all inducers (adenosine diphosphate, collagen, and arachidonic acid) except ristocetin. Based on these epidemiologic and clinicobiologic data, the diagnosis of constitutional BSS was established in six patients, while only one case was idiopathic acquired BSS.

Conclusions: Our study demonstrated that BSS is a thrombopathy that can be either constitutional or acquired, with a relatively high prevalence in Morocco, and should not be underestimated.

## Introduction

BSS is a rare platelet disorder first described by Professors Jean-Bernard and Jean-Pierre Soulier in 1948 [1]. Its worldwide prevalence is estimated at approximately one in one million [2]. BSS is characterized by an impairment in platelet adhesion, resulting from a quantitative or qualitative deficiency of glycoprotein (GP) Ib-IX-V, the receptor for von Willebrand factor (vWF). This deficiency leads to prolonged bleeding and an absence of platelet aggregation in response to the ristocetin agonist in vitro [3].

The genetic abnormalities underlying BSS have been identified primarily in the genes encoding GPIbα, GPIbβ, and GPIX, located on chromosomes 17, 22, and 3, respectively [4,5]. However, to date, no cases of BSS linked to an isolated mutation in the GPV gene have been reported in the literature [6]. Although most BSS cases are inherited in an autosomal recessive manner, often associated with consanguinity, cases of autosomal dominant transmission as well as acquired forms have also been documented [7,8]. Clinically, BSS manifests as severe mucocutaneous bleeding of variable intensity, which can sometimes be life-threatening [9]. The most common clinical manifestations include bilateral epistaxis, gingival bleeding, menorrhagia, and excessive, prolonged bleeding after trauma. Less commonly, gastrointestinal bleeding may occur [10]. These symptoms typically present in early childhood. However, BSS is often misdiagnosed due to its rarity and symptomatic overlap with other hemostatic disorders. This is particularly concerning in developing countries where diagnostic resources may be limited [5].

Biologically, patients with BSS have a prolonged bleeding time, which can exceed 20 minutes, with a normal or moderately decreased platelet count [11]. The presence of macrothrombocytes or even giant platelets is a key feature observed in blood smears [12]. Platelet aggregation studies show a normal response to ADP, collagen, and arachidonic acid (AA) but no aggregation in response to ristocetin, which is characteristic of the syndrome [4,13].

In this study, we focus on the diagnosis of BSS using platelet aggregometry in the Moroccan epidemiologic context, conducted at the Central Laboratory of Hematology of Centre Hospitalo-Universitaire Ibn Sina in Rabat.

## Materials and methods

We conducted a retrospective and descriptive study of patients diagnosed with BSS in our laboratory over a four-year period from August 15, 2020, to August 15, 2024. The study was based on complete blood count results and platelet aggregometry analyses performed on platelet-rich plasma (PRP). Prior to laboratory testing, each patient underwent a detailed clinical interview that included the collection of epidemiologic and clinical data, such as age, sex, bleeding type, and a personal history of bleeding events. Additionally, a family history assessment was conducted to evaluate the presence of consanguinity as a potential contributing factor.

Preanalytical phase

Three blood samples were collected from each patient: one EDTA tube for complete blood count and two tubes containing 0.109 M trisodium citrate (3.2%) at a citrate/blood ratio of 1:9 for platelet aggregation testing. To ensure the accuracy of the results, patients who had received platelet transfusions, aspirin, non-steroidal anti-inflammatory drugs, or steroids within 10 days prior to testing were excluded from the study. In addition, blood samples were collected after a rest period to minimize the effects of physical activity and were analyzed within two hours of collection.

Analytical phase

Platelet Count

Platelet counts were measured using a Beckman Coulter DxH 900 automated analyzer (Beckman Coulter, Inc., Brea, CA, USA). Platelet aggregation testing was performed only in patients with platelet counts greater than 80 G/L.

Platelet Aggregometry

APACT 4004 platelet aggregometry (LABiTec® Labor BioMedical Technologies GmbH, Ahrensburg, Germany) is a functional test specific to primary hemostasis. It is based on the ability of platelets to aggregate in the presence of an exogenous agonist at a temperature of 37°C. Two main methods are used: impedance aggregometry and light transmission aggregometry. In our study, we focused on the latter method because our laboratory is equipped with an APACT 4004 aggregometer, which operates based on variations in light transmission. This test was performed on PRP in the presence of four physiological agonists: ADP, collagen, AA, and ristocetin.

Before starting the test, the aggregometer was turned on for 20 minutes to reach physiological conditions, specifically a stable temperature of 37°C. Next, initial centrifugation was performed at 200-250 g for 10 minutes at 20°C without braking or by sedimentation if giant platelets were present. A second centrifugation was then performed at 3500 g for 15 minutes to collect PPP, which was used as a blank (100% transmittance) and diluent. The platelet count of the PRP is measured using a Beckman Coulter DxH 900. The target is 300 G/L.

When the platelet concentration of the PRP exceeds the desired value, it is diluted with PPP according to the following formula: $C_i \times V_i = C_f \times V_f$, where Ci is the initial PRP concentration, Vi is the volume of PPP used for dilution, Cf is the final PRP concentration (set at 300 G/L), and Vf is the final volume (set at 2000 µL). Finally, 225 µL of PRP is added to the four wells of the aggregometer, and 25 µL of agonists are added to each well (ADP at 10 μM, collagen at 5 μg/mL, AA at 0.5 mg/mL, and ristocetin at 1.5 mg/mL). The aggregometric profile develops over 5 to 10 minutes and is then compared to that of a healthy control.

## Results

Of the 268 platelet aggregation assays, seven patients had an aggregometric profile compatible with BSS. The mean age was 21 years, with a median of 10 years (range, 5-87 years). There was a male predominance, with five patients being male and two being female. Consanguinity was found in six cases.

Clinically, six patients had a history of early childhood bleeding (epistaxis, gingivorrhagia, ecchymosis), whereas only one patient presented with trauma-free hemarthrosis at an advanced age (87 years), suggesting acquired BSS. All the demographic, clinical, and biological data are shown in Table 1.

**Table 1:** Demographic, clinical, and biological characteristics of patients with BSS BSS: Bernard-Soulier syndrome, SD: standard deviation, ADP: adenosine diphosphate, AA: arachidonic acid

Characteristic	Number of patients (n=7)	Percentage (%)	Additional information
Demographic data			
Age (years)			
Mean (± SD)	21 (± 29.6)	-	-
Median (range)	10 (5-87)	-	-
Sex			
Male	5	71.4	-
Female	2	28.6	-
Consanguinity	6	85.7	-
Clinical data			
Early childhood bleeding	6	85.7	Epistaxis, gingivorrhagia, ecchymosis
Late-onset hemarthrosis	1	14.3	Atraumatic bleeding
Biological data			
Normal hemostasis parameters	7	100	-
Microcytic hypochromic anemia	4	57.1	-
Thrombocytopenia	6	85.7	-
Thrombocytosis	1	14.3	-
Presence of macroplatelets on blood smear	7	100	-
Platelet aggregation testing			
Response to ADP, collagen, AA	7	100	Normal response
Response to ristocetin	0	0	Absent response

**Figure 1:**
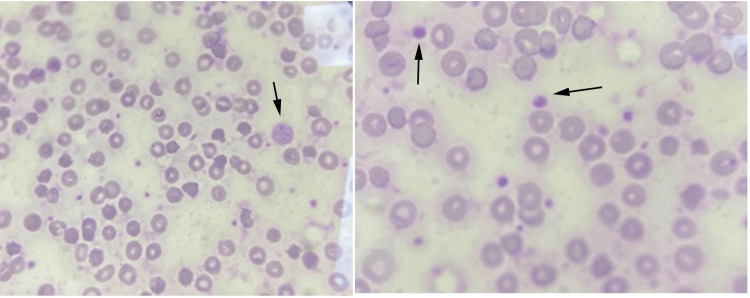
Blood smear from a BSS patient showing the presence of macro platelets and a giant platelet (arrow) (x1000) BSS: Bernard-Soulier syndrome

Biologically, all patients had normal hemostasis parameters. Microcytic hypochromic anemia was observed in four cases. All showed thrombocytopenia except for one case in which thrombocytosis was observed in an 87-year-old patient. Blood smears from all patients revealed the presence of macroplatelets (Figure 1).

**Figure 2:**
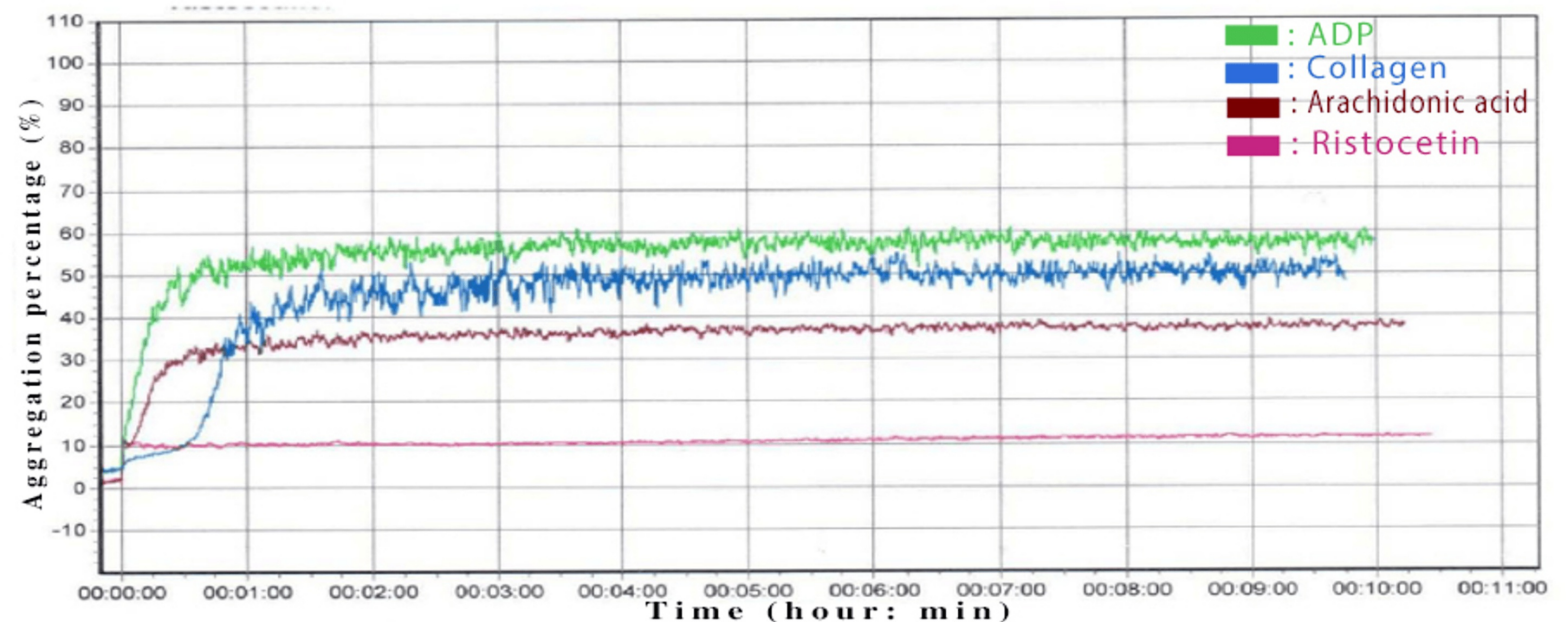
Platelet aggregation profile of a patient with BSS Green: ADP (10 μm), blue: collagen (5 μg/mL), red: AA (0.5 mg/mL), pink: ristocetin (1.5 mg/mL) BSS: Bernard-Soulier syndrome, ADP: adenosine diphosphate, AA: arachidonic acid

Platelet aggregation testing showed a normal response to all inducers (ADP, collagen, AA) except ristocetin (Figure 2) (Table 1).

Based on these epidemiologic and clinicobiologic data, six patients were diagnosed with constitutional BSS. One case was classified as idiopathic acquired BSS.

## Discussion

During the study period, seven cases of BSS were diagnosed. In the Moroccan context, a similar study conducted in our laboratory between March 2011 and September 2013 identified only one case [14]. In contrast, an Egyptian study conducted over a 16-year period reported seven cases of BSS [15]. Moreover, a retrospective study conducted at a tertiary care hospital in Lahore, Pakistan, between 2006 and 2013 documented 49 cases of BSS [16].

Furthermore, some consensus estimates put the prevalence of BSS at one per million; to date, barely a hundred cases have been reported in the medical literature, reflecting the rarity of this disease [17]. However, it is important not to exclude the possibility that the prevalence is much higher than currently estimated due to under-recognition and misdiagnosis.

Because the disease is often unrecognized, it is rarely diagnosed early in life, and the mean age of diagnosis is 16 years [17]. In our study, the mean age was 21 years with a median of 10 years, suggesting a delay in diagnosis, probably due to the unavailability of platelet aggregation tests in the Kingdom's laboratories. It should be emphasized that our laboratory, as a reference laboratory, receives patients from all over Morocco. Regarding the gender distribution, a male predominance was observed in our study. However, since BSS is inherited through autosomal genes, it affects both sexes equally [18,19]. It is also important to note that BSS is frequently observed in certain ethnic groups where consanguinity is prevalent, especially in India [20]. As consanguineous marriages are part of the Moroccan culture, this explains why the majority of patients diagnosed in our study were from consanguineous marriages [14].

In terms of clinical signs, epistaxis (60%), gingivorrhagia (20%), and ecchymosis (20%) were the most commonly reported manifestations in our study, which is consistent with the literature [21]. Intracranial and gastrointestinal bleeding are rarely reported in the literature. No such cases were observed in our study; however, joint bleeding was noted in a single patient.

Biologically, BSS is characterized by the presence of macroplatelets and normal platelet count or thrombocytopenia, which is consistent with the results of our study. Additionally, patients with BSS typically have a balanced hemostasis profile. Thus, a normal hemostasis workup does not rule out a hemostasis disorder. Another characteristic feature is the prolongation of the bleeding time, which can exceed 20 minutes. However, we did not use this technique in our study because of its potentially invasive nature, especially in the context of hemorrhagic disorders.

In our study, we relied on three main criteria to interpret platelet aggregation profiles: the slope of the aggregation curve, the maximum percentage of platelet aggregation, and a careful visual analysis of the tracings. A strong platelet response is typically defined as aggregation above 80%, while a minimal response is defined as aggregation below 20%. We used platelet aggregometry, a functional test that measures platelet aggregation by detecting changes in light transmission through PRP. Our patients' platelets responded normally to common agonists, such as ADP, collagen, and AA, indicating intact pathways of platelet activation. However, there was a complete absence of aggregation in response to ristocetin, which is a key characteristic of BSS. Ristocetin-induced aggregation tests the interaction between platelet GP Ib and vWF. In BSS, this interaction is impaired due to a deficiency or dysfunction of the GPIb-IX-V complex. Importantly, adding normal plasma did not restore the response to ristocetin, confirming that the defect lies in the platelets themselves rather than in plasma vWF. This specific aggregation pattern distinguishes BSS from von Willebrand disease, in which the ristocetin response typically improves with the addition of normal plasma [13,22].

Flow cytometric analysis of platelet GP is required for the diagnosis of BSS [23]. However, due to the unavailability of this test in our laboratory, it could not be performed on these patients. His limitation restricted our ability to identify the mutation responsible for the syndrome, differentiate between partial or complete deficiencies, and characterize the various genetic subtypes.

Genetically, this thrombopathy is primarily of constitutional origin; however, it can also be acquired through the formation of antibodies against the GP Ib-IX-V complex. In such cases, the risk of bleeding is much higher than in typical cases of immune thrombocytopenia due to the reduced platelet count combined with a significant impairment of their adhesive function [24]. This is illustrated by our patient, who developed hemarthrosis and excessive bleeding at an advanced age, leading to the diagnosis of idiopathic acquired BSS.

Our study has several limitations that should be acknowledged. Firstly, frequent delays in establishing the diagnosis were observed, sometimes occurring several years after the initial symptoms appeared. This can compromise therapeutic management and negatively affect the clinical outcome. Secondly, long-term clinical and biological follow-up was not systematically ensured, which limited the assessment of the natural progression of the syndrome, hemorrhagic complications, and treatment response. Furthermore, the absence of comprehensive family data, particularly the systematic screening of relatives, restricted our ability to analyze hereditary transmission patterns and identify asymptomatic or mildly symptomatic cases. Finally, our protocol's lack of flow cytometry and genetic analysis prevented us from formally confirming GP abnormalities and characterizing the syndrome at a molecular level.

Despite these limitations, our study has several significant strengths. Although the sample size is small, it provides valuable insight into diagnosing BSS in the Moroccan context, where data are scarce. The relatively high frequency of cases observed in our study may be attributed to the high rate of consanguinity in the studied population. This underscores the importance of more systematic screening in high-risk settings. Our study also emphasizes that although BSS is usually congenital, acquired forms have been reported, highlighting the need for greater clinical awareness. Furthermore, it demonstrates that a normal standard hemostasis workup does not exclude a platelet function disorder and that specialized investigations, such as platelet aggregometry, are essential when clinical suspicion remains. In our resource-limited setting, where advanced diagnostic tools such as flow cytometry and genetic testing were unavailable, aggregometry was the key diagnostic method. This highlights the diagnostic value of aggregometry in low-resource settings and underscores the relevance of our findings for similar healthcare contexts.

## Conclusions

Our study emphasizes that BSS is a rare yet significant thrombopathy that exists in constitutional and acquired forms. Despite its rarity, it appears to be prevalent in our setting, particularly in regions where consanguinity is common. This emphasizes the importance of considering BSS when diagnosing unexplained bleeding disorders. Platelet aggregometry is a crucial diagnostic tool that enables the accurate identification and differentiation from other platelet function disorders. Raising awareness of BSS among healthcare professionals, particularly those working with high-risk populations, could lead to earlier recognition of the disorder, more timely interventions, and improved outcomes for patients affected by it. Furthermore, our study emphasizes the need for broader, multicenter epidemiological research to determine the true prevalence and genetic diversity of BSS in our country. Such data would support the development of targeted screening strategies and healthcare planning in regions with elevated genetic risk.
